# Spectral characterization of the impact of modifiers and different prepare temperatures on snow lotus medicinal residue-biochar and dissolved organic matter

**DOI:** 10.1038/s41598-024-57553-6

**Published:** 2024-04-11

**Authors:** Sha Zhang, Zenghong Sun, Yanna Yao, Xinyu Wang, Shuge Tian

**Affiliations:** 1 College of Traditional Chinese Medicine, Xinjiang Medical University, Ürümqi, 830017 Xinjiang China; 2 Xinjiang Tianshan Lotus Medicine (Co., Ltd.), Changji, 831500 Xinjiang China

**Keywords:** Snow lotus medicine residue, Biochar, Dissolved organic matter, Two-dimension correlation spectroscopy, Excitation-emission-matrix spectra, Analytical chemistry, Environmental chemistry, Green chemistry, Materials chemistry, Medicinal chemistry, Surface chemistry, Biogeochemistry

## Abstract

This study involved the production of 20 biochar samples derived from secondary medicinal residues of Snow Lotus Oral Liquid, processed within the temperature range of 200–600 °C. Additionally, four medicinal residues, including dissolved organic matter (DOM), from 24 samples obtained using the shaking method, served as the primary source material. The investigation focused on two key factors: the modifier and preparation temperature. These factors were examined to elucidate the spectral characteristics and chemical properties of the pharmaceutical residues, biochar, and DOM. To analyze the alterations in the spectral attributes of biochar and medicinal residues, we employed near-infrared spectroscopy (NIR) in conjunction with Fourier-infrared one-dimensional and two-dimensional correlation spectroscopy. These findings revealed that modifiers enhanced the aromaticity of biochar, and the influence of preparation temperature on biochar was diminished. This observation indicates the stability of the aromatic functional group structure. Comparative analysis indicated that Na_2_CO_3_ had a more pronounced structural effect on biochar, which is consistent with its adsorption properties. Furthermore, we utilized the fluorescence indices from UV–visible spectroscopy and excitation-emission-matrix spectra with the PARAFAC model to elucidate the characteristics of the fluorescence components in the DOM released from the samples. The results demonstrated that the DOM released from biochar primarily originated externally. Aromaticity reduction and increased decay will enhance the ability of the biochar to bind pollutants. Those results confirmed the link between the substantial increase in the adsorption performance of the high-temperature modified charcoal in the previous study and the structural changes in the biochar. We investigated the structural changes of biochar and derivative DOM in the presence of two perturbing factors, modifier and preparation temperature. Suitable modifiers were selected. Preparation for the study of adsorption properties of snow lotus medicinal residues.

## Introduction

Chinese medicines have responded to advancements in modern science and technology, liberating themselves from the constraints of traditional pharmaceutical methods and diversifying their pharmaceutical dosage forms. Consequently, Chinese medicine has garnered increasing recognition both domestically and internationally. Amid the rapid progress of Chinese medicine, issues arising from its popularity have become increasingly conspicuous and cannot be overlooked. Notably, challenges, such as the high cost of treatment, environmental pollution, and resource wastage resulting from the accumulation of Chinese medicine residues, have gained prominence. The annual production of Chinese medicine residues has reached nearly 70 million tons, making the management of this issue a focal point within the realm of traditional Chinese medicine^1,2^.

Biochar, a carbon-rich substance produced through anaerobic treatment at either low or high temperatures, is gaining widespread use owing to its multifaceted porosity and remarkable adsorption characteristics. Considering the substantial yield and cost of disposal for Chinese medicine residues, the production of biochar from these residues constitutes a vital approach for environmentally friendly utilization. This approach represents a novel direction for the judicious exploitation of residues resources by converting waste medicinal residues into charcoal adsorbents capable of secondary use in the adsorption of wastewater, heavy metals, and other deleterious pollutants^3,4^. The efficacy of biochar in adsorbing pollutants is intricately linked to the raw material of biochar, the type and quantity of internal functional groups, its porosity, and other defining characteristics^5^. Existing research typically employs physical or chemical methods to modulate the structural and property changes of biochar, thereby enhancing its adsorption of heavy metals and other deleterious pollutants from wastewater^6^.

Dissolved organic matter (DOM), an offshoot of biochar, plays a pivotal role in determining its potential applicability in environmental remediation. DOM is considered a significant active chemical constituent of ecosystems, constituting organic matter that is soluble in water or acid/base solutions. It encompasses compounds, such as dissolved organic carbon, organic nitrogen, and organic phosphorus^7^. DOM exhibits mobility and reactivity within soil and water, and participates in diverse ecological processes. There are two categories of DOM: endogenous and exogenous, with exogenous DOM having residual origins, and endogenous DOM resulting from human activities^8,9^. This differentiation can alter the content and attributes of DOM within its original environment, potentially yielding both positive and negative effects. Consequently, the characterization of biochar-derived DOM serves as an essential evaluation criterion for biochar applications^10,11^.

Snow Lotus Herb (Aussureae involucratae herb) is a precious traditional Chinese medicine cultivated in the northwestern region of China and has significant medicinal value in clinical practice^12^. Snow Lotus Oral Liquid, a singular prescription formulation derived from Snow Lotus Herb, has been used in the treatment of various forms of rheumatism and rheumatoid arthritis. Even after the extraction of Snow Lotus Herb, the residual Snow Lotus residues retain vital components such as polysaccharides and flavonoids, underscoring their relevance in traditional Chinese medicine^13^. It is evident that traditional Chinese medicine continues to offer practical benefits. Consequently, methods involving active ingredient enrichment, composting substrate utilization, newborn fuel creation, biochar production, and other forms of reuse have progressively become integral to the sustainable development of residues resources. This transition holds immense significance for extending the overall value of these resources^14,15^.

Previous studies confirmed that high temperature and alkali modification could enhance the adsorption of methyl orange and methyl red to 480 mg/g and 720 mg/g^16^. In order to further investigate the effect of biochar by high temperature and modifiers, we conducted a comparative analysis of the structural transformations between residues and biochars, considering two influencing factors: distinct chemical modifiers and various calcination temperatures. The samples were prepared using three alkaline solutions (Na_2_CO_3_, K_2_CO_3_, and NaOH) as modifiers at five temperature settings. Biochar obtained from unmodified dregs served as the control. Preliminary analysis of the sample spectral characteristics was performed using infrared spectroscopy and correlation analysis. To investigate the effects of two disturbing factors, different chemical modifiers and different preparation temperatures, on the structure of pharmaceutical residue and biochar. Based on the observed structural changes in the residues and biochar itself, along with the fluorescence characteristics of the released DOM, preference was given to modifier selection. This choice laid the foundation for subsequent investigations of the adsorption mechanism of biochar. The alterations in the structural attributes of biochar and its derivatives, in turn, serve as a theoretical basis for the systematic exploration of novel avenues for harnessing the secondary residues of snow lotus oral liquid^17–19^.

## Materials and methods

### Materials

Secondary medicine residues of Snow Lotus Oral Liquid (Xinjiang Tianshan Lotus Medicine Co., Ltd., China), Sodium Carbonate (w/v ≥ 99.6%), Potassium Carbonate (w/v ≥ 99.0%), Sodium Hydroxide (w/v ≥ 96.0%), Hydrochloric Acid (12 mol/L), distilled water, ultrapure water.

### Instruments

XS-105 analytical balance, from Meller Toledo, Switzerland. Swing type Chinese medicine crusher (Model: AK-1000A, China Wenling Aoli Chinese Medicine Machinery Co). Vacuum drying oven (DZF-6051 Beijing Yongming Medical Instrument Co., Ltd., China). Vacuum tube furnace (OTF-1200X-S, Hefei Kejing Material Technology Co., Ltd., China). IRPrestige-21 Shimadzu Fourier Transform Infrared Spectrometer (SHIMADZJ, Japan). High Power CNC Ultrasonic Cleaner (Model: KQ-200KDE, Kunshan Ultrasonic Instruments Co., Ltd., China). Near-infrared spectrometer (Ocen Optics Spectrsuite, USA); pH meter (PHSJ-3F, Remagnetics, Shanghai, China). Constant temperature shaking chamber (Model: OMW-44, Changsha Kilon Instrument Co., China). UV–Visible Spectrophotometer (Model: UV-2700, Shimadzu, Japan). Fluorescence spectrophotometer (Model: RF-6000, Shimadzu, Japan).

### Preparation of pharmaceutical residues and biochar samples

To prepare the raw material for biochar production, an appropriate amount of Snow Lotus Oral Liquid residue was taken and decocted twice in 10 times the volume of water. The first decoction lasted 1 h, followed by a second decoction for 30 min. After filtration, the remaining secondary residues were dried and passed through a 150–180 mesh sieve. This material was designated as T1.

To create unmodified biochar, the secondary residues powder of Snow Lotus was dried to a constant weight, and then placed in a vacuum tube furnace with nitrogen ventilation for a slow pyrolysis process lasting 2 h. The pyrolysis was conducted at temperatures of 200 °C, 300 °C, 400 °C, 500 °C, and 600 °C, with a temperature increase rate of 5 °C/min and a nitrogen flow rate of 50 mL/min. After cooling, the resulting product was labeled as secondary residue biochar (SBC200–SBC600 °C), yielding five distinct samples, all stored in a desiccator.

To prepare the modified samples, the initial powder was soaked in a 10% solution of Na_2_CO_3_, K_2_CO_3_, or NaOH (1:8 ratio) for 24 h. Subsequently, they were dried to a constant weight. Three distinct modified residue powders were labeled TA (activated secondary residues by Na_2_CO_3_), TK (activated secondary residues by K_2_CO_3_), and TN (activated secondary residues by NaOH). Pyrolysis was performed following the procedure described in the second paragraph of "Preparation of pharmaceutical residues and biochar samples" section. The resulting modified charcoal was rinsed with 0.1 mol HCl and distilled water until the pH reached 7–8. Fifteen samples were designated as SBA200–600 °C (activated secondary residue biochar by Na_2_CO_3_), SBK200–600 °C (activated secondary residue biochar by K_2_CO_3_), and SBN200–600 °C (activated secondary residue biochar by NaOH). The samples were stored in a desiccator.

In this study, the 24 samples were categorized into four groups: SBC as the control group and SBA, SBK, and SBN as experimental groups, with different modification methods for intergroup comparison. Within each group, intra-group comparisons of the spectral features were conducted at different burning temperatures ranging from 200 to 600 °C. Meanwhile, the yield of the biochar in each group was calculated according to Eq. (1).

1 $${\text{Yield}}=\frac{{{\text{M}}}_{2}}{{{\text{M}}}_{1}}\times 100\mathrm{\%}$$

In the formula, M_2_ represents the mass of the calcined biochar and M_1_ is the mass of the sample before calcination.

#### Fourier transform infrared spectroscopy characterisation

The Fourier transform infrared spectroscopy (FT-IR) encompassing 21 samples of T1 and the 4 groups of biochar were acquired using the KBr pressing method, spanning the wavelength range of 400–4000 cm^−1^. Additionally, the NIR spectral features for each sample were captured using an NIR spectrometer (Ocen optics Spectrsuite) over a wavelength range of 800–2400 nm. These spectral data, along with FT-IR first-order spectroscopy, were analyzed using Origin 2022 in conjunction with the 2D-COS method.

In this study, the scorching temperature and modification method of biochar were employed as external perturbation factors to investigate their impact on the biochar structure. Using 2D-COS analysis, the original spectral signals were expanded by one dimension to generate 2D correlation spectra for the pharmaceutical residue biochar^20–22^.

The computation of the 2D correlation spectra involves the application of the Hilbert-Noda transform to dynamic spectra, resulting in synchronous (*Φ*) and asynchronous (*Ψ*) correlation spectra:

Synchronous spectrum formula:

2 $$\Phi \left({\upnu }_{1},{\nu }_{2}\right)=\frac{1}{N-1} \sum_{j=1}^{N}\widetilde{y}({\nu }_{1 },{p}_{j})\widetilde{y}({\nu }_{2},{p}_{j})$$

Asynchronous spectral formula:

3 $$\psi \left({\upnu }_{1},{\nu }_{2}\right)=\frac{1}{N-1} \sum_{j=1}^{N}\widetilde{y}{(\nu }_{1 },{p}_{j})\sum_{k=1}^{N}{M}_{jk}\cdot \widetilde{y}({\nu }_{2},{p}_{k})$$

In these formulas, *Φ*(ν_1_,ν_2_) denotes the alteration in the similarity of the spectral intensities at ν_1_ and ν_2_ with respect to the p value, whereas* Ψ*(ν_1_, ν_2_) signifies the modification in phase anisotropy. In Eqs. (3) and (4), ${M}_{jk}$ denotes the elements situated at line j and column *k* within the Hilbert-Noda transformation matrix.

4 $${M}_{jk}=\left\{ \begin{array}{ll}0 & \quad if \; j=k \\ \frac{1}{\pi (k-j)} \\ & \quad otherwise\end{array}\right.$$

Following Noda's law, the automatic fronts and cross peaks within each group of samples were derived by integrating the attributes of the one-dimensional spectra of these samples with the synchronization and asynchronization provided by the 2D-COS method. This approach enables the assessment of the sequential relationship between changes in infrared spectral intensities both among and within groups of samples when subjected to perturbing factors^23^.

### Extraction of DOM from samples

DOM was extracted using the constant-temperature shaking method. This involved weighing 1 g of the four types of dregs and biochar powder and mixing them with 100 ml of ultrapure water. The mixture was sealed and placed in a constant temperature shaker set at 25 °C and 180 r·min^−1^, where it was shaken for 24 h. Subsequently, it was centrifuged at 4000 r·min^−1^ for 20 min, and the resulting supernatant was filtered through a 0.45 μm aqueous filtration membrane. This filtered solution was obtained as drug residue and biochar DOM solution, which was then stored in a refrigerator at 4 °C. The numbering and grouping of the samples were consistent with the method described in "Preparation of pharmaceutical residues and biochar samples" section.

#### Ultraviolet spectral characterization of sample DOM

The Ultraviolet Spectral (UV) absorbance values serve as indicators of the chemical structural traits of DOM. We employed a Shimadzu UV-2700 spectrophotometer to assess the absorption spectra of DOM derived from the raw powder of the medicine residue and biochar. Samples lacking UV absorption after biochar treatment at temperatures exceeding 500 °C were excluded. Multiple spectral characteristics were computed for each group of samples to determine alterations in the chemical attributes of DOM.

Ultrapure water was employed as the blank calibration baseline, and a spectral range of 200–800 nm was chosen to acquire the absorbance values (A) and subsequently compute the absorption coefficient.

5 $${\alpha }_{g }(\lambda )=2.303A/{\text{r}}$$

where ${\alpha }_{g }(\lambda )$ is the absorption coefficient (m^−1^) of, stands for the wavelength (nm), and r is the optical path (m).

A_254_ corresponds to the absorbance at 254 nm, and the higher absorbance value indicates the larger relative molecular mass of the DOM. UVA_254_ represents the ratio of the absorbance coefficient to the dissolved organic carbon concentration at 254 nm, which characterizes the changes in DOM's aromatic structure of the DOM. The change in UVA_254_ was directly proportional to the degree of DOM aromatization. A_254_/A_203_ provides insight into the type of substituents on the aromatic rings within DOM. If the aromatic ring is substituted with hydroxyl and amino groups, this ratio increases; conversely, if the aromatic ring is substituted with carboxyl and carbonyl groups, the ratio decreases. A_254_/A_203_ can also indicate the type of aromatic ring substituents in DOM, where an increase suggests hydroxyl and amino group substitutions and a decrease implies carboxyl and carbonyl group substitutions. A_254_/A_365_ characterizes the size of DOM molecules, A_300_/A_400_ reflects the degree of DOM degradation, and A_465_/A_665_ represents the content of proteins and carbohydrates within the DOM^24^.

S represents the slope of the UV spectral curve and the magnitude of the S value is inversely related to the molecular weight of DOM. This parameter can be used to gauge the size of DOM molecules and provide insight into photochemical reactions and other related information. To calculate the corresponding spectral slopes S_275-295_ and S_350-400_, two wavelength bands, 275–295 nm and 350–400 nm, were selected. These slopes were used to depict the changes in DOM characteristics. The calculation formula is as follows:

6 $$\alpha (\uplambda )=\alpha ( {\uplambda }_{\upgamma }){{\text{e}}}^{[{\text{S}}({\uplambda }_{\upgamma }-\uplambda )]}$$

where $\mathrm{\alpha }(\uplambda$) is the absorption coefficient at the measurement wavelength and $\mathrm{\alpha }$(${\uplambda }_{\upgamma })$ is the absorption coefficient at a reference wavelength of 440 nm.

The S_R_ represents the ratio of the slopes of the S_275-295_ and S_350-400_ spectra. It is frequently employed to characterize structural changes in DOM. The S_R_ value was independent of the concentration and inversely proportional to the molecular weight of DOM. A low molecular weight DOM indicates exogenous or recently generated DOM, whereas a high molecular weight suggests predominantly endogenous DOM or a strong bleaching effect^25^.

7 $${\text{S}}_{{\text{R}}} = {\text{S}}_{{{275} - {295}}} /{\text{S}}_{{{35}0{-}{4}00}}$$

#### DOM characterization of samples by fluorescence index

The fluorescence index (*FI*) is defined as the ratio of the fluorescence intensity when Ex is 370 nm and Em is 470 and 520 nm. This index provides insight into the contribution of aromatic and non-aromatic amino acids to the fluorescence intensity of DOM, shedding light on the source and degradation of DOM. An *FI* value below 1.4 suggests that the sample DOM primarily originates from terrestrial or soil sources. An *FI* between 1.4 and 1.9 indicates that the sample DOM results from a combination of exogenous and endogenous sources, while an FI exceeding 1.9 suggests the presence of microbial influences.

The autogenous source index (*BIX*) was calculated as the ratio of fluorescence intensity at Ex 245 nm to Em at 380 nm and 430 nm. This index reflects the proportion of endogenous DOM within a sample relative to the overall DOM content. A *BIX* greater than 1 indicates that the endogenous DOM is predominantly influenced by microorganisms in the sample, where as a *BIX* ranging from 0.6 to 0.7 suggests that the DOM primarily originates from terrestrial sources or human activities.

The decay index (*HIX*) is defined as the ratio of the average fluorescence intensity of Em within the range of 435–480 nm and 300–345 nm when Ex is set at 245 nm. The *HIX* provides information on the degree of decay of the DOM sample^26^. An *HIX* value less than 4 indicates a low degree of DOM decay, whereas an *HIX* between 10 and 16 suggests a high degree of DOM decay^27^.

#### EEMs and PARAFAC analysis of biochar’s DOM

The samples were extracted following the procedure outlined in "Preparation of pharmaceutical residues and biochar samples", and the fluorescence spectrophotometer (Shimadzu RF-6000) was employed for sample analysis. Ultrapure water was used as the blank solution. Excitation and emission wavelengths were scanned across the ranges of 200–750 nm and 250–800 nm, respectively, with a step size of 5 nm and a scanning speed of 30,000 nm-min^−1^. A 1 cm quartz fluorescence cuvette was used as the vessel.

To process the data, Rayleigh scattering and Raman scattering were removed using Matlab software and DOMflour software packages. This was performed to establish the PARAFAC model identification. The stability of the model was assessed through split-half and residual analyses to predict the fluorescence components of the samples after anomalous data were removed. The fluorescence profiles of the DOM samples were further processed and plotted using the MATLAB 2018 software. The PARAFAC model was calculated using the following formula:

8 $${x}_{ijk}=\sum_{f=1}^{F}{a}_{if}{b}_{jf}{c}_{kf}+{e}_{\begin{array}{c}ijk\end{array}}$$

where *χ*_*ijk*_ represents the fluorescence intensity of *i* sample point at excitation wavelength* k* and emission wavelength *j*. *a*_*if*_ represents the factor score, which signifies the proportion of the concentration of the *f* component relative to the concentration of *i* sample. where *b*_*jf*_ and *c*_*kf*_ represent the loadings, which denote the relative values of the *j* emission and *k* excitation spectra, respectively, with respect to the *f* component. *e*_*ijk*_ corresponds to the residual element and *F* is the number of component factors defined within the model. where *F* is the number of component factors in the model^28^.

## Results and discussion

### Biochar samples yield

Following the computation of the biochar yield for each group, it was observed that the experimental group generally exhibited a higher biochar yield than the control group (Table 1).

**Table 1:** Yields of four biochar species at different preparation temperatures (n = 3 ${\overline{\text{x}}}$ ± s).

Temp (℃)	Category			
	Yield (%)			
	SBC	SBA	SBK	SBN
200	95.77 ± 0.77	98.13 ± 0.56	96.75 ± 0.60	98.68 ± 0.06
300	94.18 ± 0.92	95.23 ± 0.43	94.60 ± 0.29	97.25 ± 0.14
400	93.23 ± 1.12	93.43 ± 0.63	93.95 ± 0.05	94.41 ± 0.25
500	92.34 ± 0.88	93.02 ± 0.74	93.95 ± 0.73	93.12 ± 0.37
600	91.48 ± 0.79	92.49 ± 0.41	93.44 ± 0.10	92.94 ± 0.13

SBC**:** secondary residue biochar, SBA**:** secondary residue biochar modified by Na_2_CO_3_, SBK**:** secondary residue biochar modified by K_2_CO_3_, SBN**:** secondary residue biochar modified by NaOH.

### NIR spectral characterization

**Figure 1:**
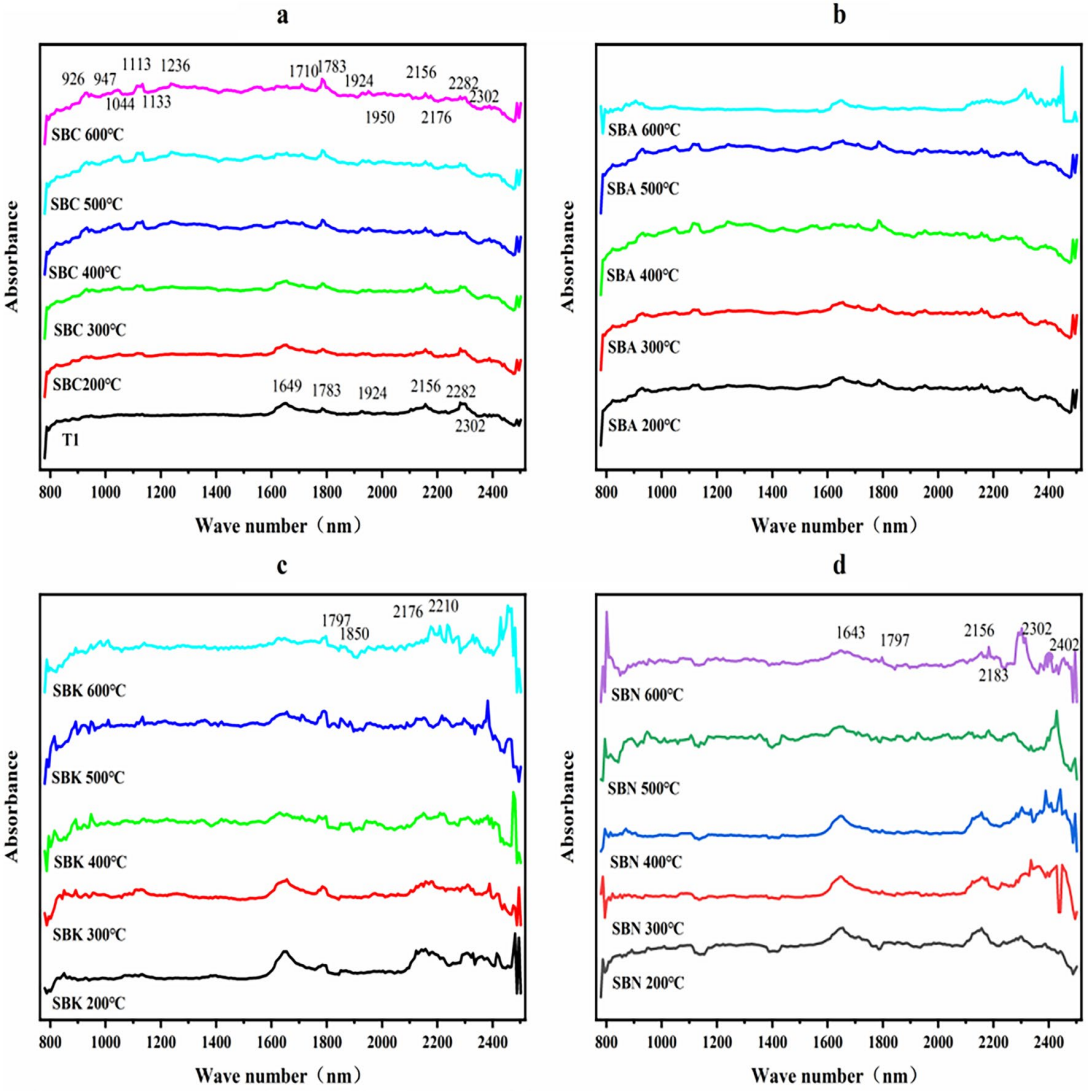
NIR absorbance spectra of four residue biochars with different pyrolysis temperature (**a** residue biochars (Tl) and secondary medicinal residue biochar (SBC) 200–600 °C, **b** secondary medicinal residue biochar activated by Na_2_CO_3_ (SBA) 200–600 °C, **c** secondary medicinal residue biochar activated by K_2_CO_3_ (SBK) 200–600 °C, **d** secondary medicinal residue biochar activated by NaOH (SBN) 200–600 °C).

The NIR spectra of the snow lotus dregs and four groups of biochar revealed the presence of two distinct characteristic absorption regions in the unmodified residue powder (T1) (see Fig. 1a). The first band, spanning from 1649 to 1924 nm, exhibited absorption peaks at 1649 nm, associated with the C-H bond absorption of the vinyl group, and at 1783 nm, likely attributed to the C–H bond absorption of the methylene group. The 1924 nm absorption is indicative of O–H bond interactions between water and polyvinyl alcohol. The second band featured absorption peaks within the range 2156–2302 nm. The peaks at 2156 nm and 2283 nm correspond to the C–H bonding characteristics of aromatic hydrocarbons, whereas the 2302 nm peak represents C–H bond absorption in the amide groups. These findings suggest the presence of aromatic, olefin, aliphatic hydrocarbon, and other functional group structures in the original medicinal residue.

Following high-temperature calcination, T1 exhibited breaking of some functional group bonds, leading to the emergence of new absorption bands within the range of 926–1236 nm. Most of these bands are associated with the C–H bond absorption characteristics of aliphatic and aromatic hydrocarbons.

In the SBA group, an increase in the oxygen-containing functional group absorption within the range of 1800–2200 nm was observed (see Fig. 1b). The SBK and SBN groups displayed C–H bond absorption characteristics of protein-like substances, amide bonds, and amide groups. Furthermore, distinctive absorptions associated with amide bonds and halogenated hydrocarbon pairs of protein-like substances were observed, along with consistent characteristics of olefinic and aromatic functional groups that remained unchanged with increasing preparation temperature (Fig. 1c and d). These results indicated that modification and high preparation temperatures increased the stability of the aromatic structures within the samples. It is also reduced the degree of temperature sensitivity of the snow lotus residues, retained the strongest structure and increased the overall toughness of the biochar.

### Characterization of FT-IR spectra

#### FT-IR one-dimensional spectral analysis

**Figure 2:**
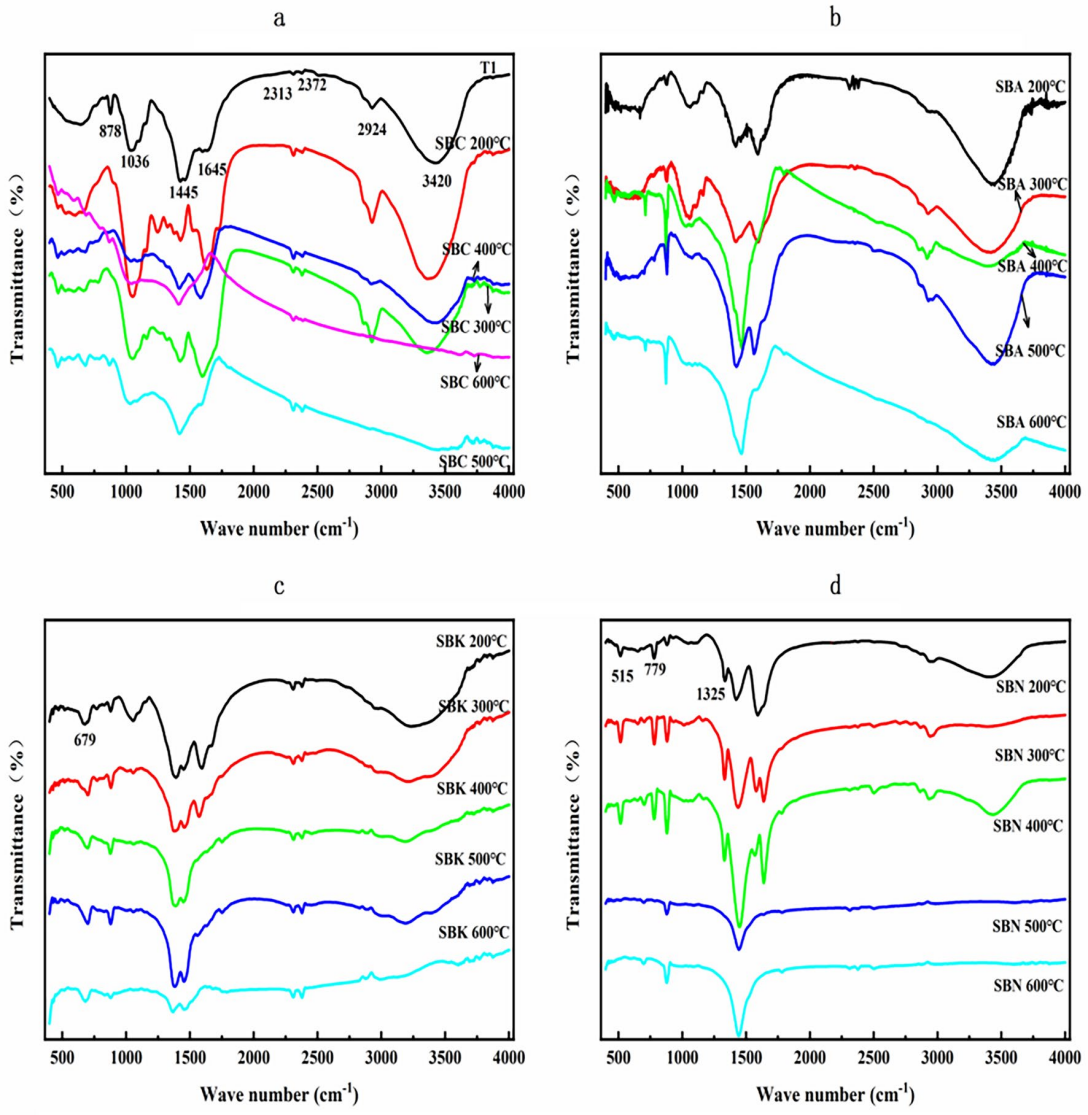
FT-IR absorbance spectra of four residue biochars with different pyrolysis temperature (**a** residue biochars (Tl) and secondary medicinal residue biochar (SBC) 200–600 °C, **b** secondary medicinal residue biochar activated by Na_2_CO_3_ (SBA) 200–600 °C, **c** secondary medicinal residue biochar activated by K_2_CO_3_ (SBK) 200–600 °C, **d** secondary medicinal residue biochar activated by NaOH (SBN) 200–600 °C).

Figure 2 illustrates the functional group characteristics within the FT-IR one-dimensional maps of snow lotus residues and biochars. The absorption peak observed at 878 cm^−1^ in T1 signifies the out-of-plane bending vibration of the C–H bond within the 1,2,4-substituted benzene. At 1036 cm^−1^, the symmetric telescopic vibration of the =C–O–C bond found in the aromatic ether can be discerned. Additionally, the absorption peak at 1445 cm^−1^ likely corresponds to the out-of-plane bending vibration of the C–C bond within the alkane or the backbone vibration of the C=C bond within the substituted benzene. Furthermore, the absorption peak at 1645 cm^−1^ is indicative of the stretching vibration of the C=C bond found in olefins, whereas the peaks at 2313 and 2372 cm^−1^ may signify the backbone vibration of the C=C bond within alkynes or the telescopic vibration peaks of the C≡C bond or the carbonyl group of alkynes. Moreover, the two absorption peaks detected at 2924 and 3420 cm^−1^ correspond to the reverse telescopic vibration of the C–H bond within alkanes and the telescopic vibration of the hydroxyl group within alcohols, respectively.

Following the modification of the residues with Na_2_CO_3_, the absorption associated with alkynes and carbonyl C–C bonds gradually disappeared. Simultaneously, the vibration of the aromatic carbon skeleton increased in tandem with an increase in the preparation temperature (Fig. 2b). Notably, the K_2_CO_3_ and NaOH-modified residues exhibited heightened absorption peaks corresponding to halogenated hydrocarbons at 515, 679, and 779 cm^−1^. When comparing Fig. 2b and d, it is evident that Na_2_CO_3_ and NaOH exerted a more significant influence on the absorption intensity of the aromatic groups at 878 cm^−1^, enhancing the absorption of aromatic functional groups. This is consistent with the conclusion of the NIR spectral characterization, which indicates that the modification can maintain the firmness of the aromatic structure of the biochar, while the high temperature preparation promotes the tendency of aromatization of the biochar structure (Fig. 2c,d).

#### FT-IR two-dimensional correlation spectrum analysis

**Figure 3:**
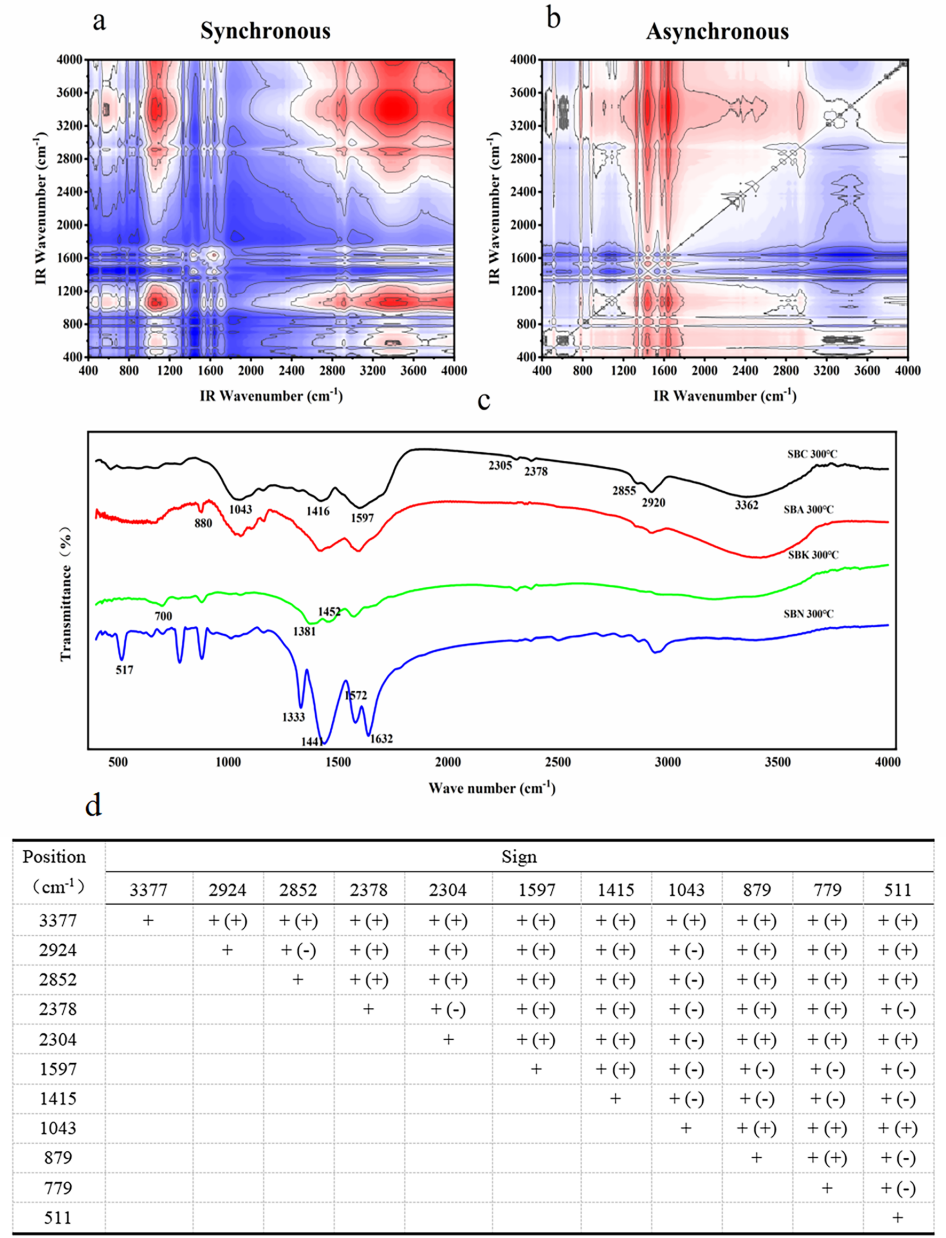
Two-dimensional correlation maps of biochars from IR spectra of the comparison between groups of modified biochar (**a** synchronous map, **b** asynchronous map, **c** one-dimensional infrared spectroscopy for comparison between modified groups and **d** sign of each cross-peak in synchronous (*Φ*) and asynchronous (*Ψ*, in parentheses) maps of modified groups biochars, + positive, − negative).

**Figure 4:**
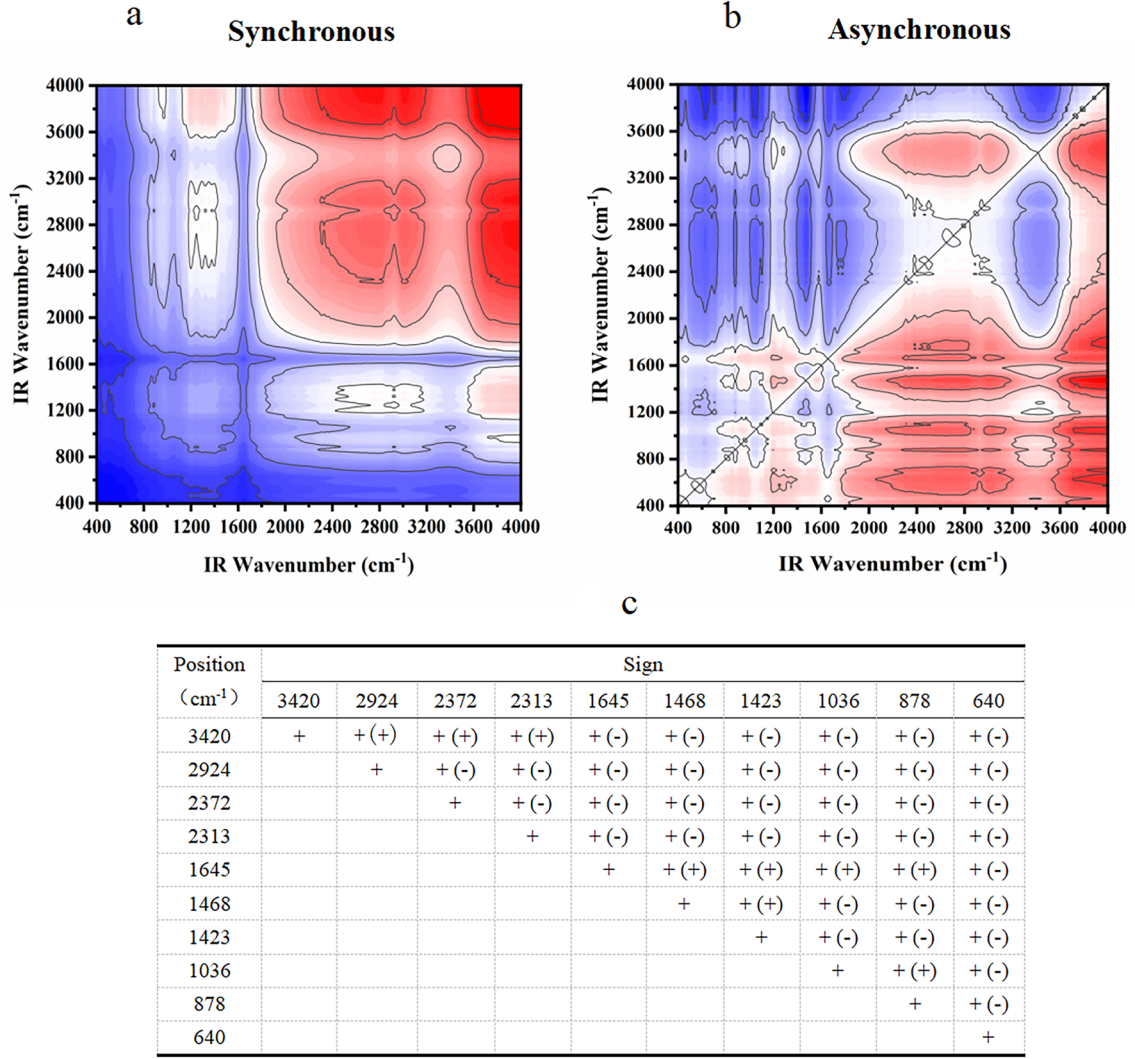
Two-dimensional correlation maps of biochars from IR spectra of the secondary medicinal residue biochar (SBC) group (**a** synchronous map, **b**: asynchronous map, and **c** sign of each cross-peak in synchronous (*Φ*) and asynchronous (*Ψ*, in parentheses) maps of SBC group biochars, + positive, −negative).

**Figure 5:**
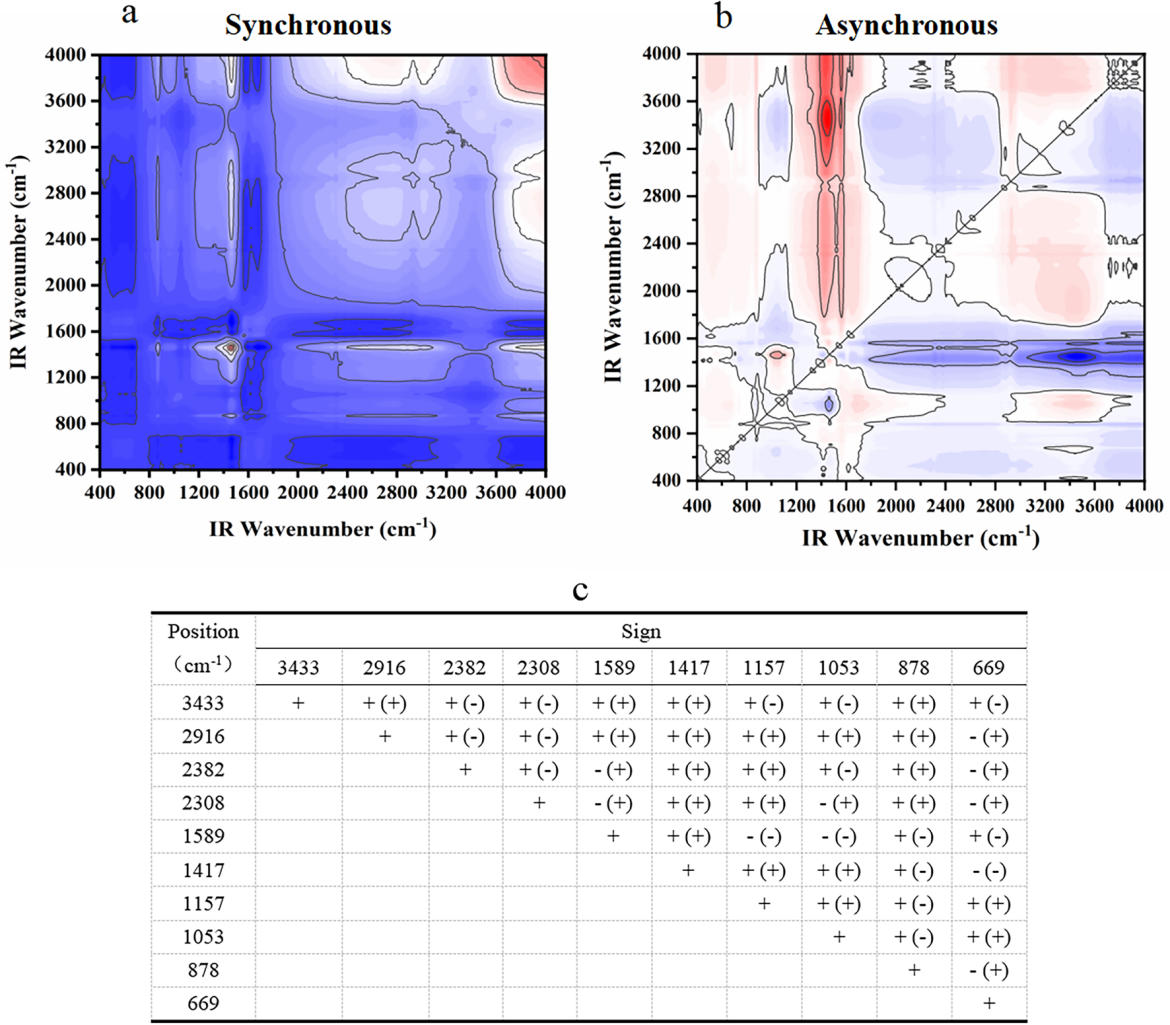
Two-dimensional correlation maps of biochars from IR spectra of the secondary medicinal residue biochar activated by Na_2_CO_3_ (SBA) group (**a** synchronous map, **b** asynchronous map, and **c** sign of each cross-peak in synchronous (*Φ*) and asynchronous (*Ψ*, in parentheses) Maps of SBA group biochars, + positive, −negative).

**Figure 6:**
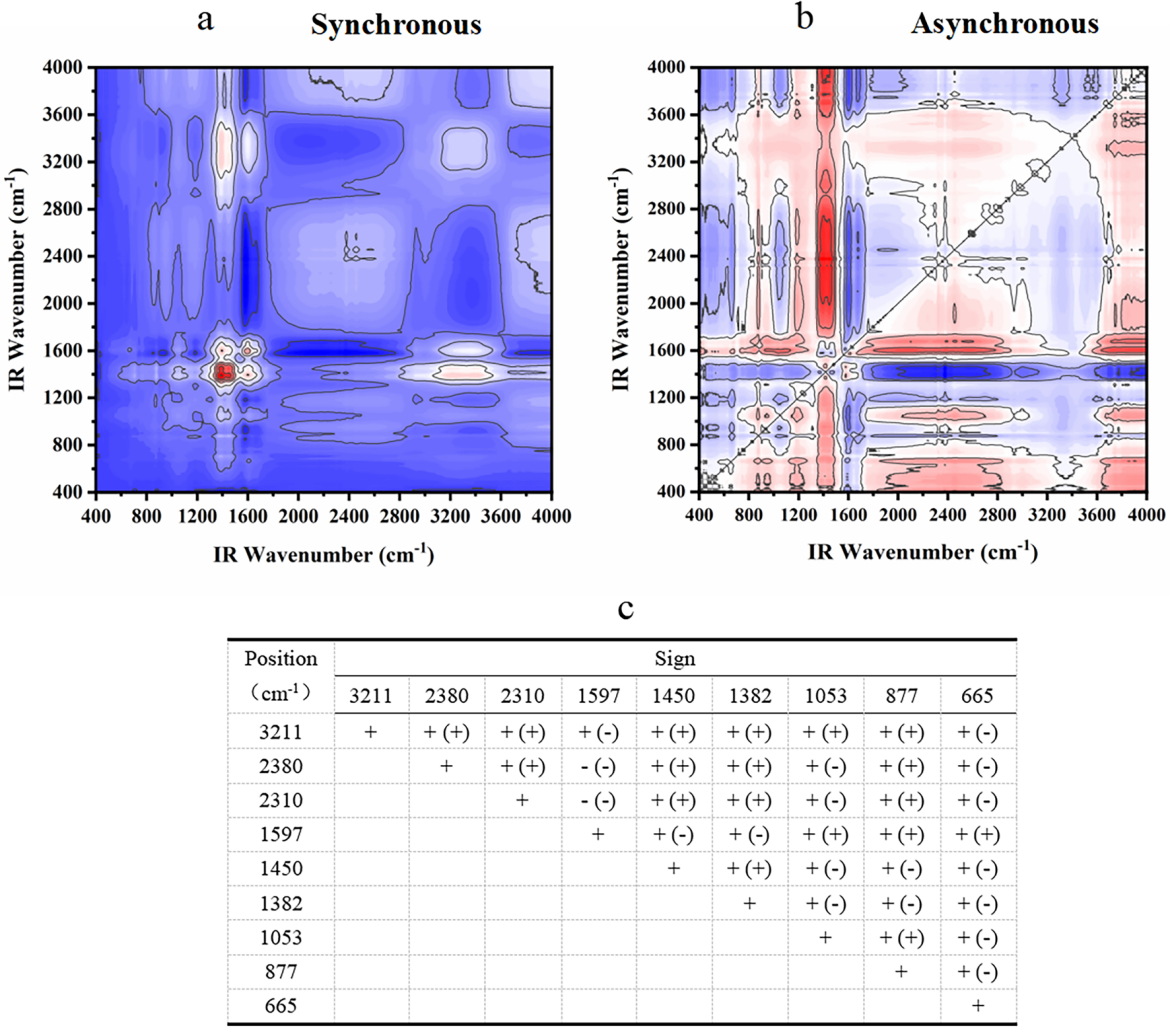
Two-dimensional correlation maps of biochars from IR spectra of the secondary medicinal residue biochar activated by K_2_CO_3_ (SBK) group (**a** synchronous map, **b** asynchronous map, and **c** sign of each cross-peak in synchronous (*Φ*) and asynchronous (*Ψ*, in parentheses) maps of SBK group biochars, + positive, −negative).

**Figure 7:**
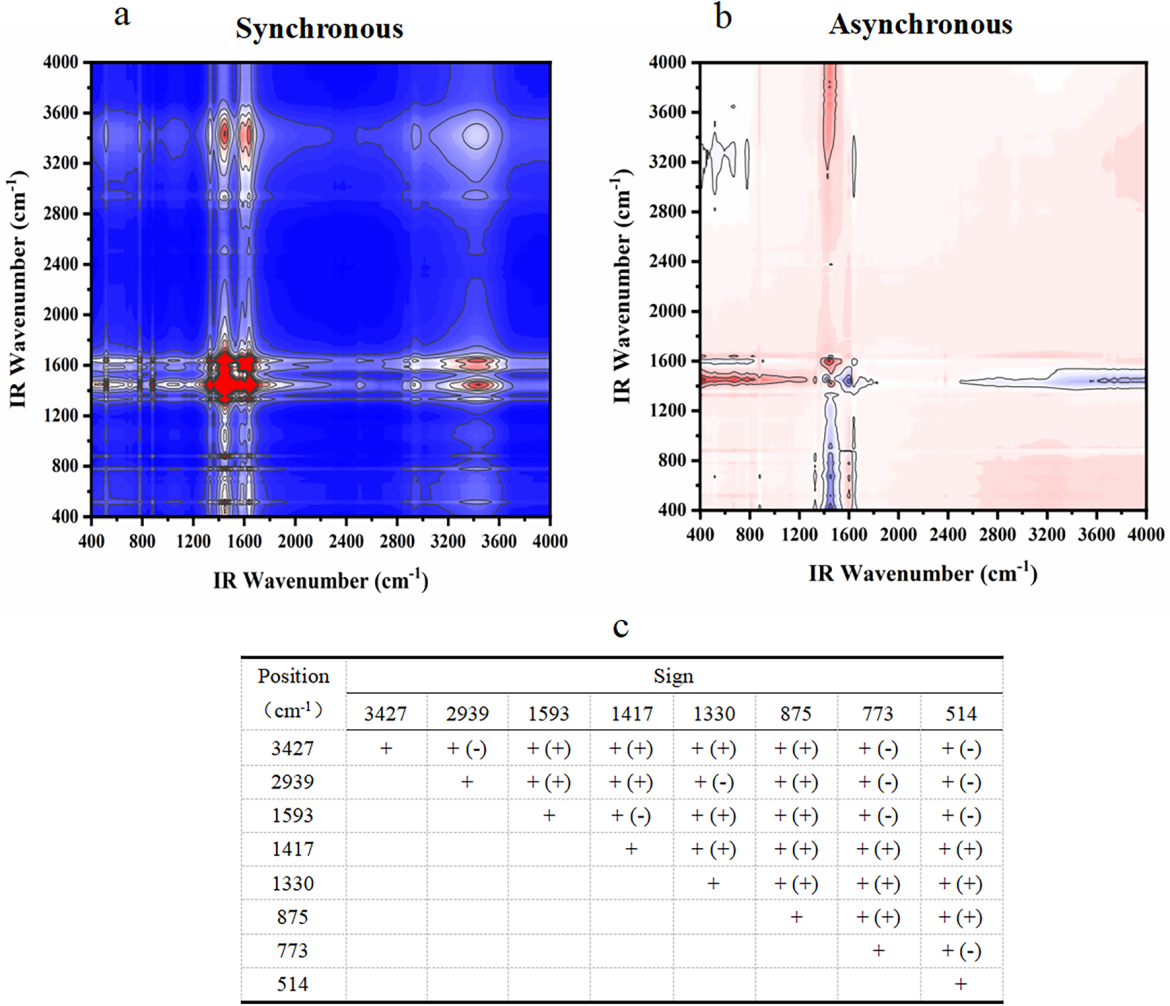
Two-dimensional correlation maps of biochars from IR spectra of the secondary medicinal residue biochar activated by NaOH (SBN) group (**a** synchronous map, **b** asynchronous map, and **c** sign of each cross-peak in synchronous (*Φ*) and asynchronous (*Ψ*, in parentheses) maps of SBN group biochars, + positive, −negative).

The 2D-COS spectra were analyzed according to Noda's law to generate the spectra and tables presented in Figs. 3, 4, 5, 6, 7. As shown in Fig. 3, the infrared absorption spectra of various residue treatments followed the order of 3377 > 1043 > 2852 > 2924 > 2304 > 511 > 2378 > 879 > 779 > 1597 > 1415 cm^−1^. Initial changes were observed in the alcohols and hydroxyl groups, with the hydroxyl groups undergoing the earliest transformation. Subsequently, changes occur in the =C–O–C bonds of aromatic ethers, C–C bonds of alkynes or carbonyls, halogenated hydrocarbons C–X, and C–C bonds of the monoskeletal surfaces within their bending vibrations. The last to undergo a change is alkanes with a C–C bond.

Figure 4 displays the spectral intensity changes within the T1 and SBC groups, showing the following sequence: 640 > 1645 > 1036 > 878 > 1468 > 1423 > 3420 > 2313 > 2372 > 2924 cm^−1^. Temperature serves as a perturbing factor for this group. The initial changes involved the out-of-plane bending vibration of the C–H bond in the olefin and aromatic hydrocarbons. Subsequent changes encompass the telescopic vibration of the C=C bond or the skeletal vibration of the benzene ring, as well as alterations in the C–H bonds on hydroxyl and unsaturated carbons. The final changes manifest as stretching vibrations of the C≡C bonds in alkynes and C–H bonds in saturated carbons.

Changes in the C–H bond bending vibrations of biochar olefins in the SBA group precede C–H bond changes on m-disubstituted benzene (669 > 878 cm^−1^). Changes in the C≡C, O–H bonds, and C–H bonds on unsaturated carbons occur between the alterations in these two groups of peaks (669 > 2308 > 2382 > 3433 > 2916 > 878 cm^−1^). Changes such as double bonds and halogenated hydrocarbons occurred later than the C–H bond changes of substituted benzenes (878 > 1589 > 1417 > 1157 > 1053 cm^−1^, as depicted in Fig. 5).

The sequence of changes in the spectral peaks within the SBK group is more intricate. The characteristics of the initial changes were similar to those observed in the SBA group. However, changes in the O–H bonds and unsaturated carbons precede those of the double bonds (665 > 1053 > 3211 > 2380 > 2310 > 877 > 1450 > 1382 cm^−1^, 1053 > 1450 > 1382 > 1597 cm^−1^, as illustrated in Fig. 6).

Within the SBN group, the spectral changes demonstrated that unsaturated carbon bonds precede double bonds and the benzene ring skeleton (3427 > 1417 > 1593 > 1330 > 875 cm^−1^, 1417 > 1593 > 1330 > 875 > 773 cm^−1^). The sequence of changes in C–H bonds on the benzene ring cannot be definitively determined, but may precede unsaturated carbon bonds (773 > 2939 > 3427 cm^−1^), along with the C–H bonds on the benzene ring (773 > 2939 > 3427 cm^−1^, as shown in Fig. 7).

It is evident that different modifications influence the order of the changes in the O–H bonds and unsaturated carbons. Simultaneously, the preparation temperature primarily affects the order of changes in the olefinic, aromatic C–H bonds, and double bonds. Notably, the three modifiers substantially enhanced the stability of aromatic functional groups within the medicine dregs and biochar, rendering them less temperature sensitive.

### UV–vis spectral index analysis of DOM

#### UV–vis characteristic absorption value

**Figure 8:**
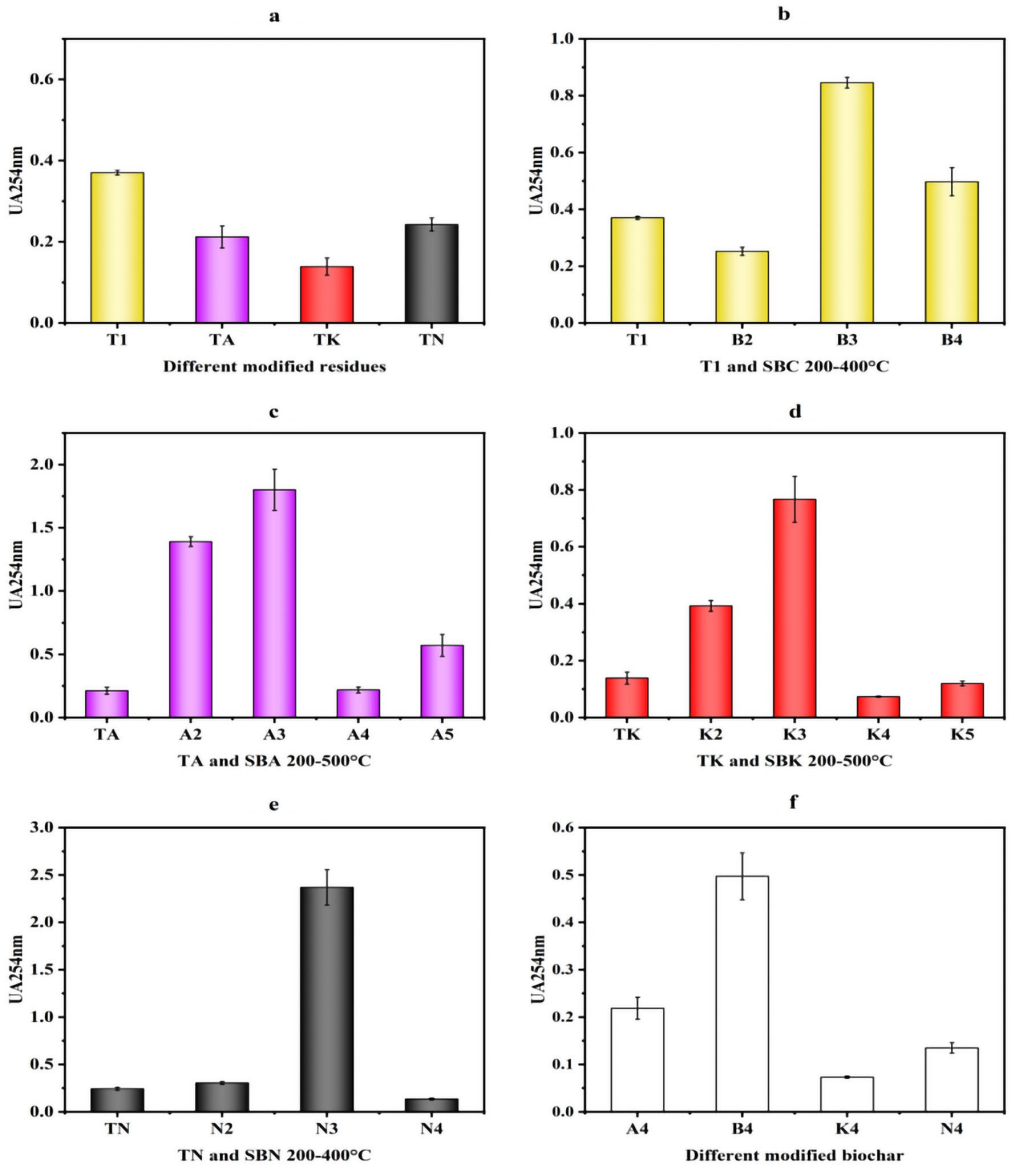
Changes in UA_254_ values of pharmaceutical residues and biochars (**a** intergroup comparison of UA_254_ values for modified drug residues, **b**–**e** intragroup comparison of UA_254_ values from comparison group and experimental groups, **f** intergroup comparison of UA_254_ values for modified biochars in same temperature).

UV_254_ was employed to characterize the structural alterations in the aromatic properties of DOM. A comparison between Fig. 8a and f reveals that both modification and preparation temperatures had the capacity to diminish the aromaticity of the pharmaceutical residue and biochar DOM.

In the context of within-group comparisons, the aromaticity of DOM within the control group and two experimental groups, SBA and SBK, displayed a consistent trend of change in response to preparation temperature. Initially, it increased and then began to decrease after reaching 300 °C. Conversely, for the SBN group, alterations in the aromaticity of DOM exhibited greater complexity (Fig. 8b–e).

Comparison of the U_250_ values of the four groups shows that modifiers and high preparation temperatures reduced the aromaticity of DOM, and the reduction of aromaticity could reduce the biotoxicity produced by biochar during adsorption and avoid secondary pollution^29^.

#### UV–vis absorption ratio

**Figure 9:**
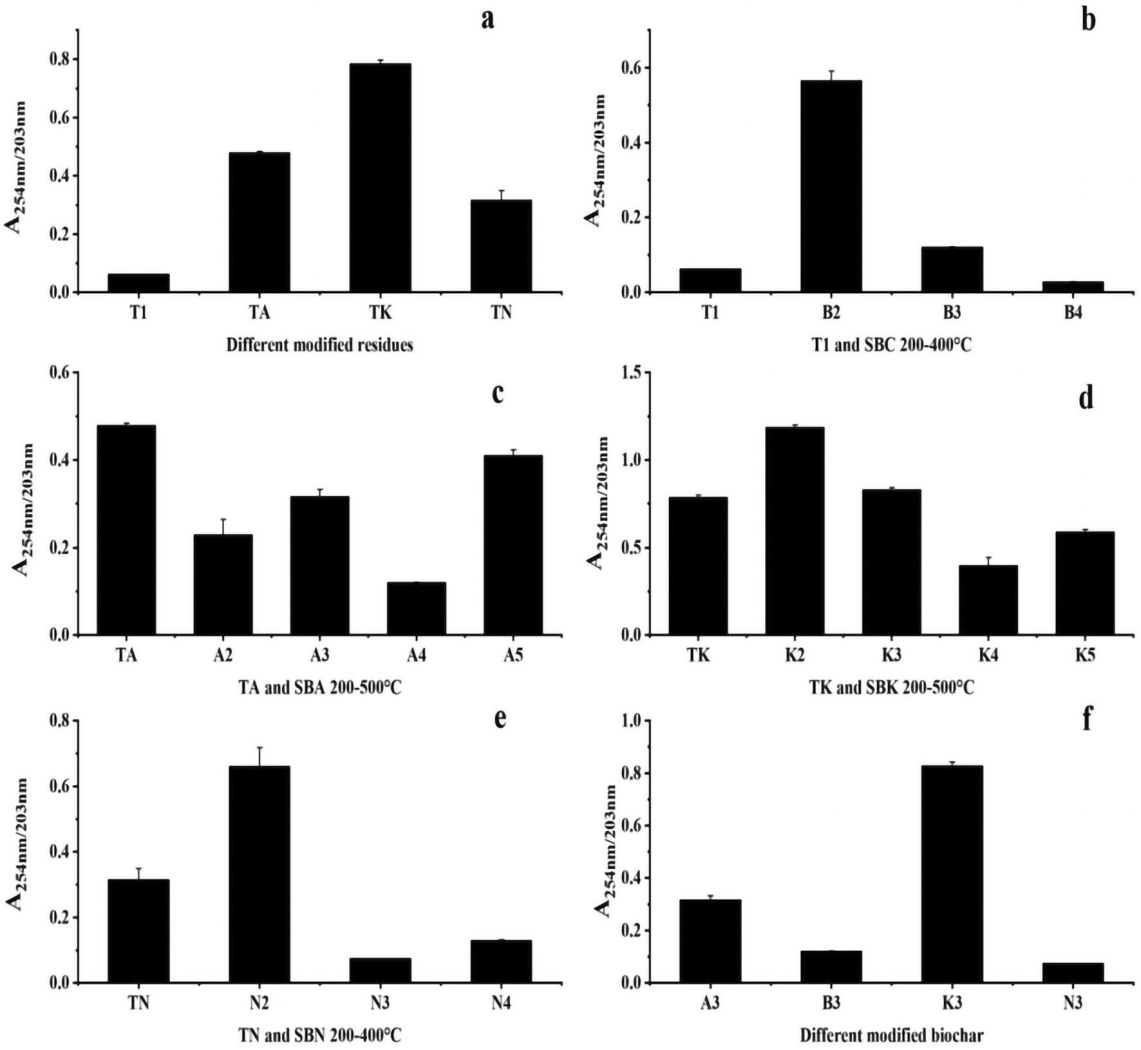
Changes in A_254_/A_203_ values of pharmaceutical residues and biochars (**a** intergroup comparison of A_254_/A_203_ values for modified drug residues, **b**–**e** intragroup comparison of A_254_/A_203_ values from comparison group and experimental groups, **f** intergroup comparison of A_254_/A_203_ values for modified biochars in same temperature).

Intergroup Comparison: The A_254_/A_203_ values, as depicted in Fig. 9a and f, serve as indicators for determining the type of substituents on the aromatic ring of DOM. The results demonstrated that Na_2_CO_3_ and K_2_CO_3_ increased the presence of aromatic rings substituted with hydroxyl and amino groups in drug residue DOM. Furthermore, the substituent aromatic rings of DOM were relatively unaffected by temperature, except for the SBN group.

**Figure 10:**
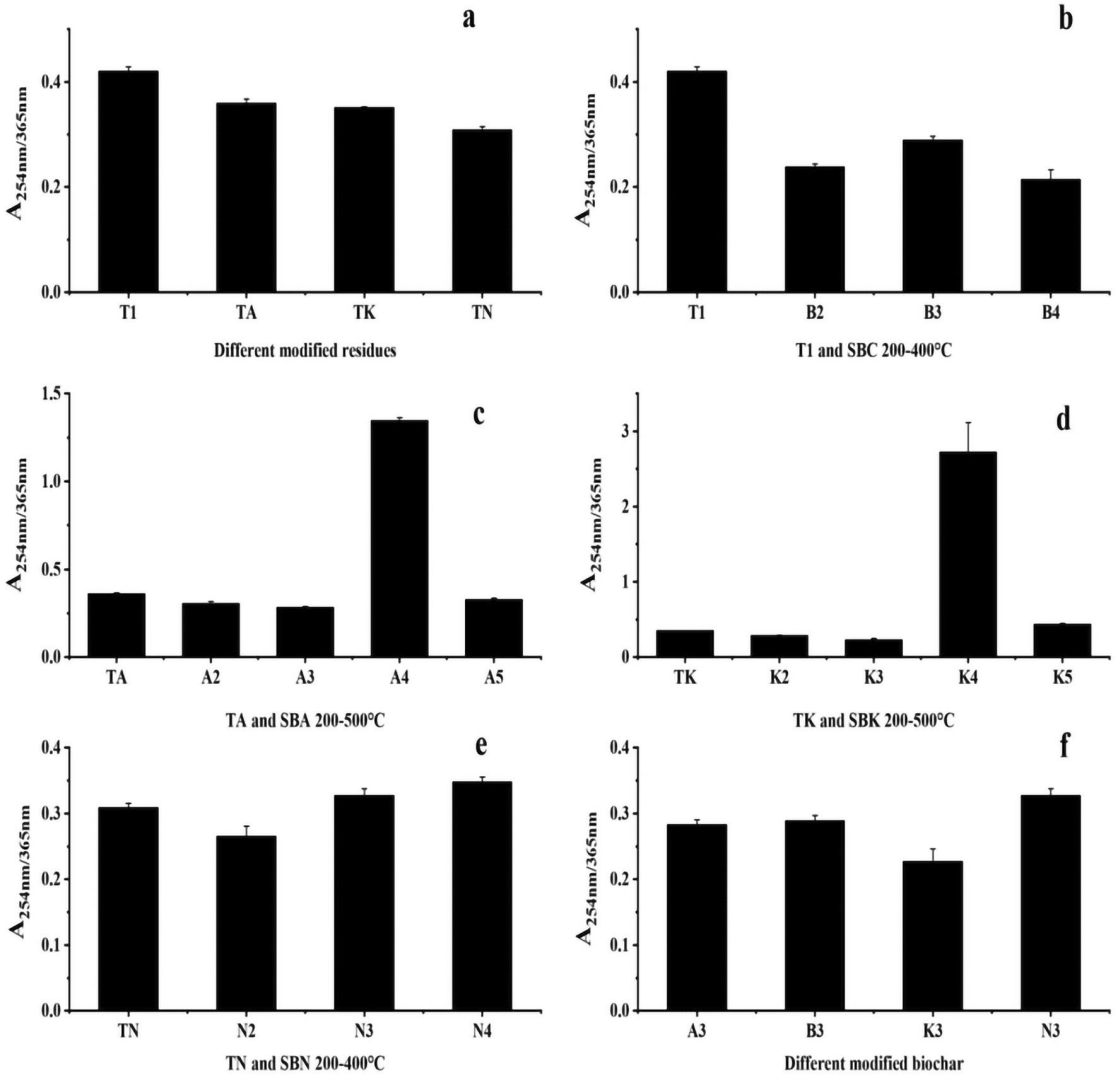
Changes in A_254_/A_365_ values of pharmaceutical residues and biochars (**a** intergroup comparison of A_254_/A_365_ values for modified drug residues, **b**–**e** intragroup comparison of A_254_/A_365_ values from comparison group and experimental groups, **f** intergroup comparison of A_254_/A_365_ values for modified biochars in same temperature).

A_254_/A_365_, used to characterize the molecular weight size of DOM, revealed that the modifiers led to a slight decrease in the molecular weight of the drug residue DOM, with no discernible influence from the preparation temperature, as shown in Fig. 10a and f.

**Figure 11:**
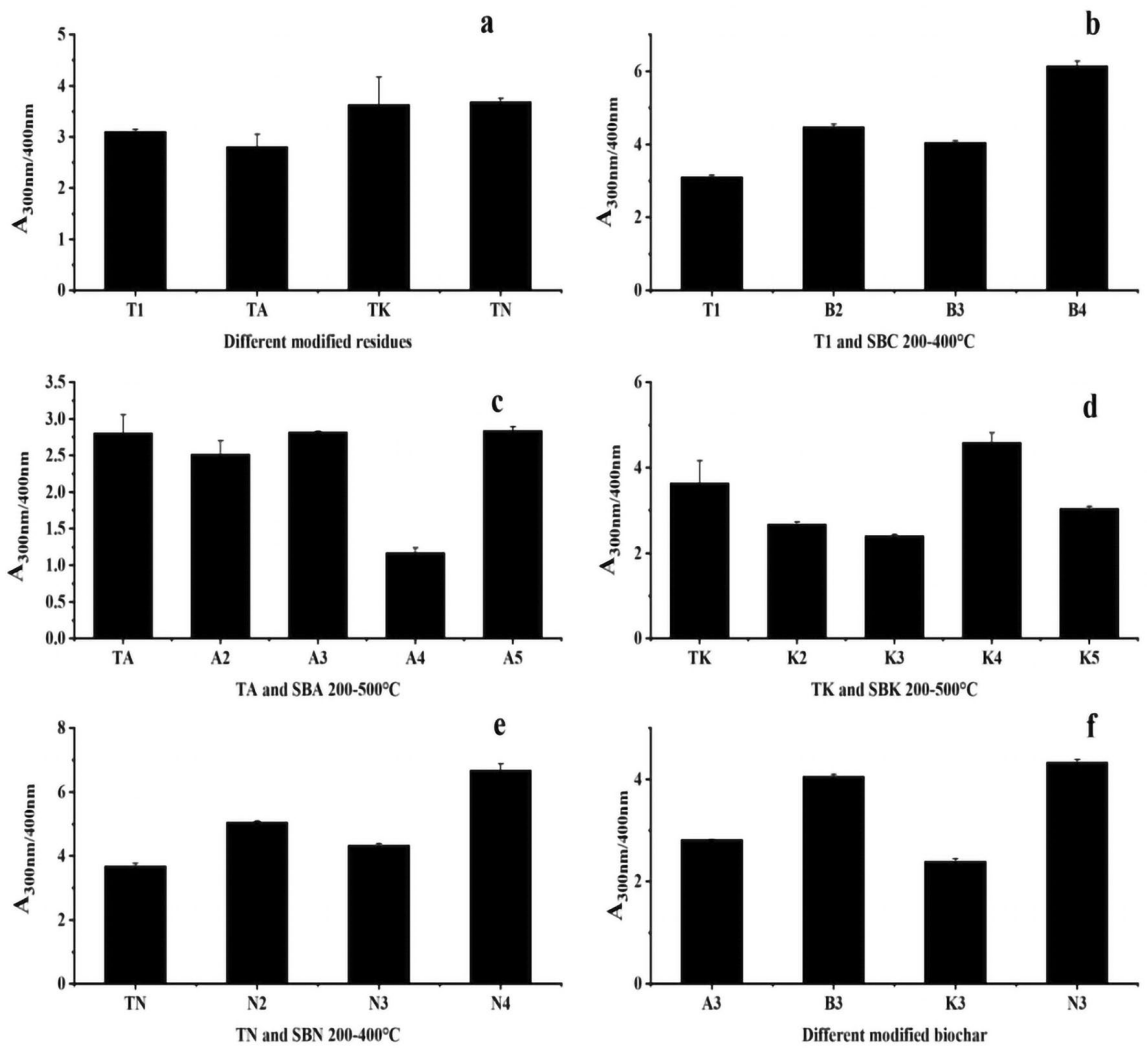
Changes in A_300_/A_400_ values of pharmaceutical residues and biochars (**a** intergroup comparison of A_300_/A_400_ values for modified dmg residues, **b**–**e** intragroup comparison of A_300_/A_400_ values from comparison group and experimental groups, **f** intergroup comparison of A_300_/A_400_ values for modified biochars in same temperature).

A_300_/A_400_, indicating the degree of DOM decay, played a role in distinguishing between humic acid and fulvic acid content. When A_300_/A_400_ < 3.5, the DOM primarily contained huminic acid, and vice versa when it was mainly fulvic acid. Figure 11a and f illustrate that the DOM of the raw drug residue powder was predominantly composed of huminic acid and remained unaffected by the modifier. After exposure to high temperatures, DOM in the control and SBN experimental groups transitioned to being primarily composed of fulvic acid.

**Figure 12:**
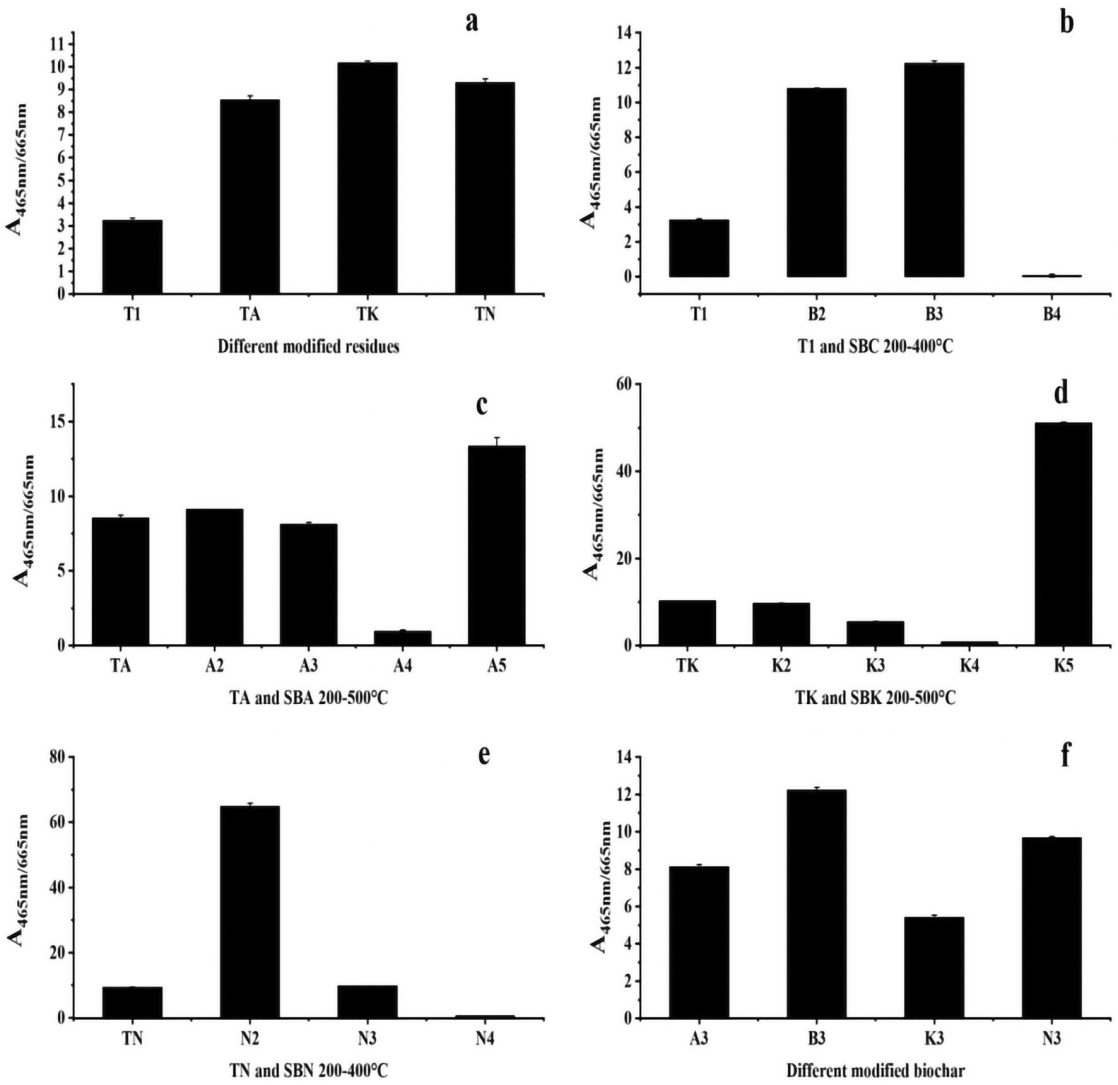
Changes in A_465_/A_665_ values of pharmaceutical residues and biochars (**a** intergroup comparison of A_465_/A_665_ values for modified dmg residues, **b**–**e** intragroup comparison of A_465_/A_665_ values from comparison group and experimental groups, **f** intergroup comparison of A_465_/A_665_ values for modified biochars in same temperature).

A_465_/A_665_, used to gauge the protein and carbohydrate contents of DOM, exhibited notable increases in protein and carbohydrate contents within the DOM of the medicine residue after modification, with an increase in carbohydrate content in the DOM of both the SBC and SBK groups in response to high temperatures, as shown in Fig. 12a and f.

Comparison Within Groups: Analysis based on Fig. 9b–e indicates that, except for the SBA group, the A_254_/A_203_ values of DOM in the other three groups displayed an increasing trend before reaching 200 °C. This suggests a higher presence of aromatic rings substituted with hydroxyl and amino groups. However, the ratio decreased as the cleavage temperature exceeded 200 °C, indicating an increase in carbonyl and carboxyl-substituted aromatic rings. In contrast, for the SBA group, the carbonyl- and carboxyl-substituted aromatic rings of DOM increased with increasing cleavage temperature.

The A_254_/A_365_ values of DOM within each group (Fig. 10b–e) indicate that in the control group, higher cleavage temperatures corresponded to lower molecular weight DOM. Conversely, the molecular weights of DOM in the SBA and SBK groups remained relatively stable across temperatures, except for a notable increase at 400 °C. In the SBN group, the molecular weight of DOM slightly increased with increasing cleavage temperature.

Intergroup comparisons of A_300_/A_400_ for DOM (Fig. 11b–e) revealed that DOM in the experimental groups was dominated by fulvic acid, except for the SBA and SBK groups.

Lastly, Fig. 12b-e demonstrates that the protein and carbohydrate contents of DOM in the control group were significantly influenced by the preparation temperature. In contrast, after the modifier intervention, the protein and carbohydrate contents of DOM remained relatively unaffected by the preparation temperature across all groups.

#### S-value vs. SR-value

**Figure 13:**
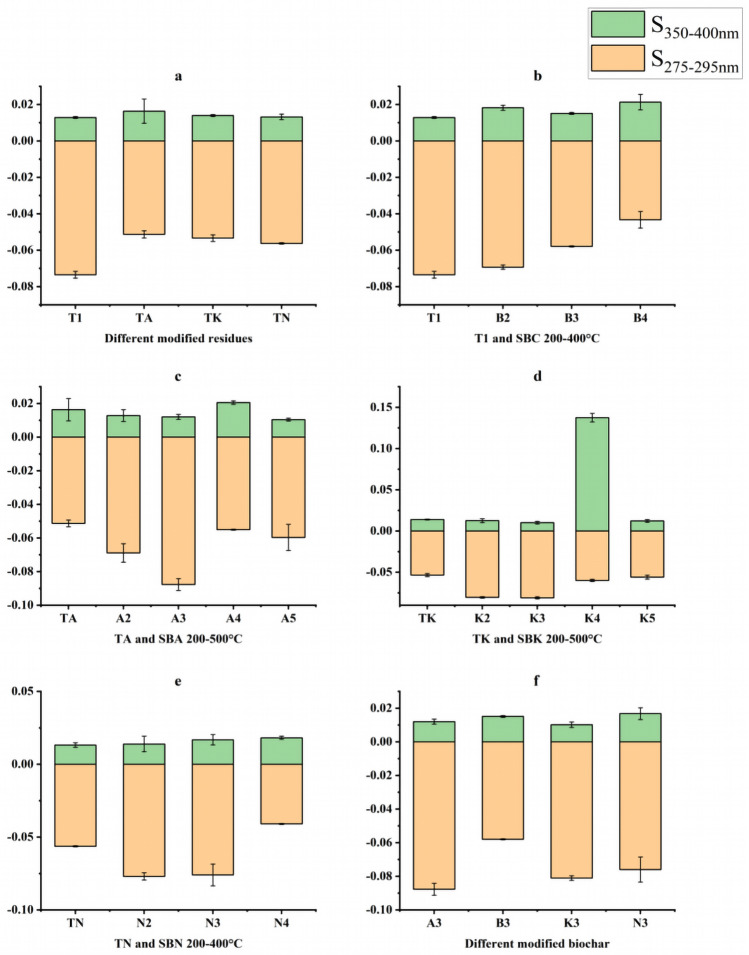
Changes in S_275-295_ and S_250-400_ values of pharmaceutical residues and biochars (**a** intergroup comparison of S_275-295_ and S_250-400_ values for modified dmg residues, **b**–**e** intragroup comparison of S_275-295_ and S_250-400_ values from comparison group and experimental groups, **f** intergroup comparison of S_275-295_ and S_250-400_ values for modified biochars at same temperature).

As depicted in Fig. 13a–f, the S_275-295_ and S_250-400_ values generally exhibit an increasing trend, indicating that both the modification method and preparation temperature contribute to a reduction in the molecular weight of DOM.

Table 2 provides insights into the S_R_ values, all of which were < 1. This observation suggests that the production of drug residues and biochar DOM in this study primarily stems from external environmental factors.

**Table 2:** S_R_ value of residues and biochars for each group (n = 3 ${\overline{\text{x}}}$ ± s).

Category	SR
T1	− 5.9010 ± 0.1253
SBC200℃	− 3.4926 ± 0.1130
SBC300℃	− 4.0223 ± 0.1430
SBC400℃	− 2.4822 ± 0.0831
TA	− 4.5732 ± 0.0554
SBA200℃	− 6.2242 ± 0.1341
SBA300℃	− 7.6435 ± 0.1728
SBA400℃	− 2.6928 ± 0.1137
SBA500℃	− 5.9743 ± 0.0551
TK	− 4.1174 ± 0.0551
SBK200℃	− 7.4152 ± 0.1395
SBK300℃	− 7.2925 ± 0.0284
SBK400℃	− 0.4169 ± 0.0340
SBK500℃	− 4.8144 ± 0.1359
TN	− 4.7543 ± 0.1669
SBN200℃	− 4.3467 ± 0.1669
SBN300℃	− 4.9798 ± 0.1669
SBN400℃	− 2.4158 ± 0.0648

T1, SBC**:** secondary residue and biochar; TA, SBA: secondary residue and biochar modified by Na_2_CO_3_; TK, SBK: secondary residue and biochar modified by K_2_CO_3_; TN,SBN: secondary residue and biochar modified by NaOH.

#### DOM fluorescence index analysis

**Figure 14:**
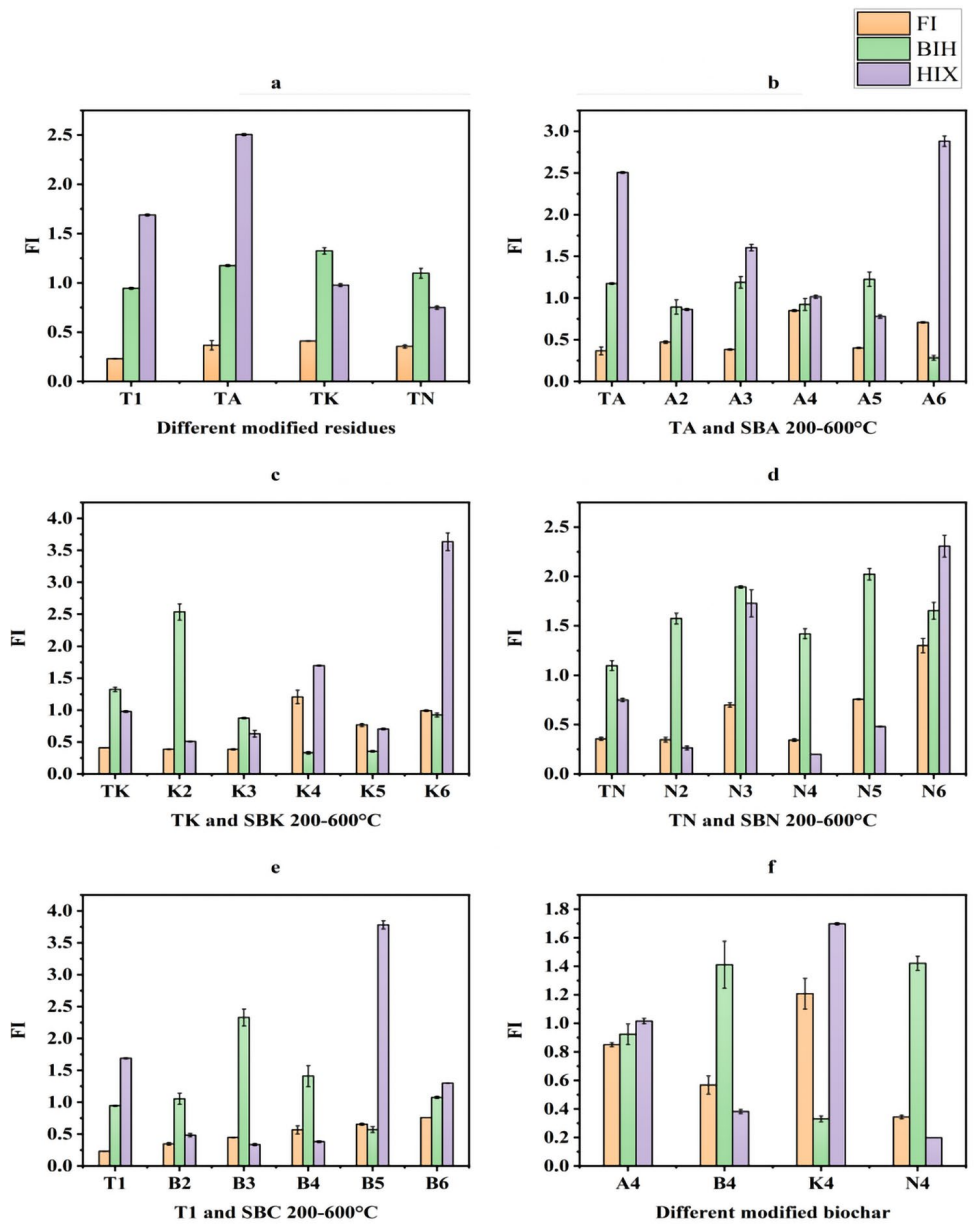
Changes in fluorescence indices of pharmaceutical residues and biochars (**a** intergroup comparison of fluorescence indices for modified drug residues, **b**–**e** intragroup comparison of fluorescence indices from comparison group and experimental groups, **f** intergroup comparison of fluorescence indices for modified biochars at same temperature).

According to Fig. 14, the *FI* values for DOM in all samples were < 1.4, indicating that the DOM in these samples likely originated from terrestrial sources, possibly soil.

A comparison of the *BIH* index results revealed that the endogenous source of DOM in the samples primarily originated from microbial influences, except for five samples: SBA600 °C and SBK300–600 °C.

Furthermore, the *HIX* values for all samples were < 4, indicating that the degree of DOM decay in the study samples was relatively low^30^.

#### 3D-EEM spectral characterization of DOM

**Figure 15:**
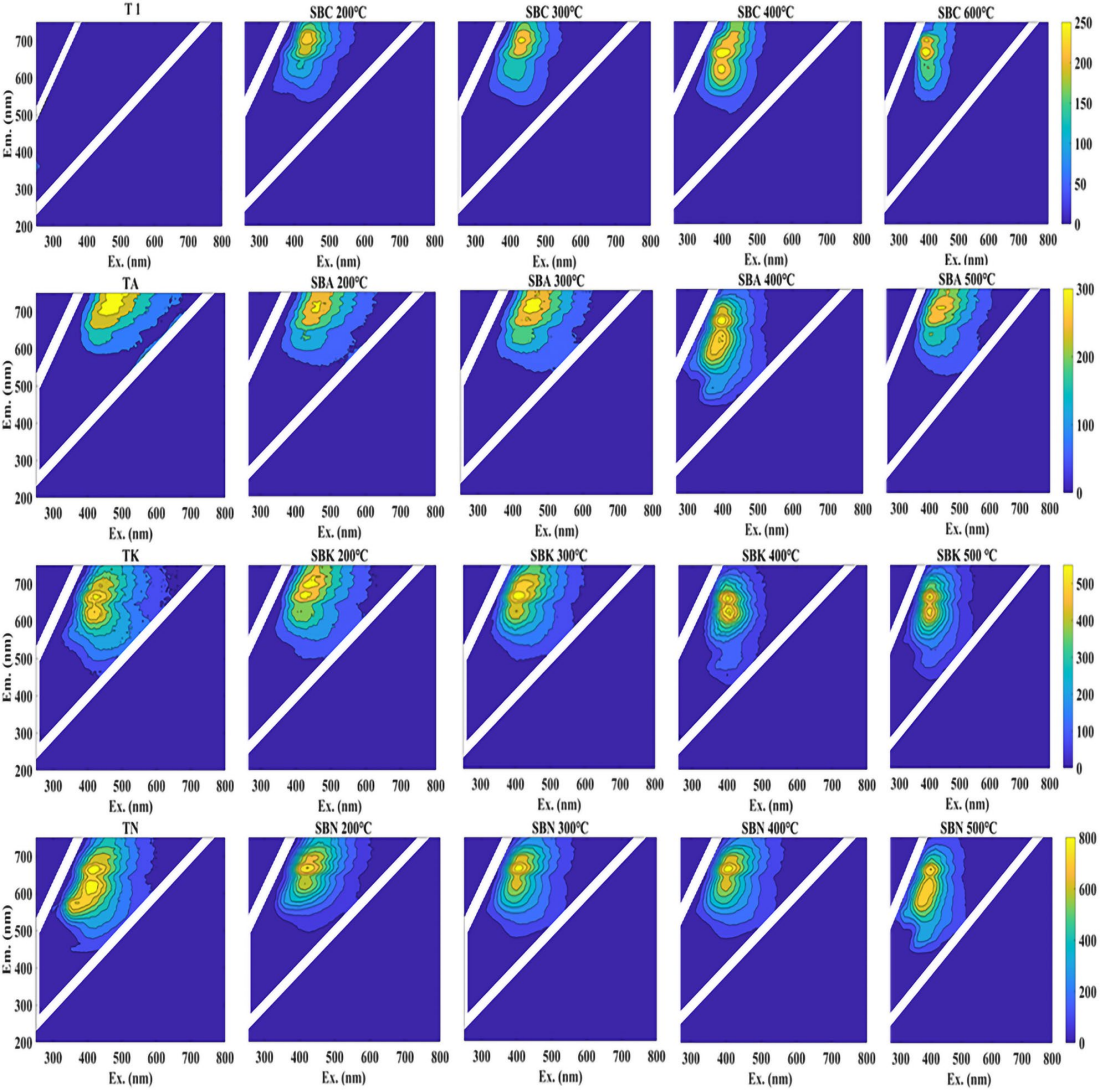
3D fluorescence mapping of DOM from pharmaceutical residues and biochars.

The samples were analyzed using a Shimadzu RF-6000 three-dimensional fluorescence spectrometer, and the three-dimensional fluorescence spectra of the samples were acquired. It is evident from the visual representation that the high-temperature modification and cracking resulted in the release of fluorescent substances. Notably, the excitation wavelengths of these fluorescent substances exhibited a slight shift towards higher wavelengths as the modification and preparation temperatures increased. This shift can likely be attributed to the modification process, which enhances the conjugated system within the sample molecules, rendering it more stable. Additionally, the preparation temperature influenced the molecular activities and intermolecular interactions, leading to alterations in the electronic energy levels and charge densities, ultimately causing shifts in the fluorescence peaks (Fig. 15^31^).

**Figure 16:**
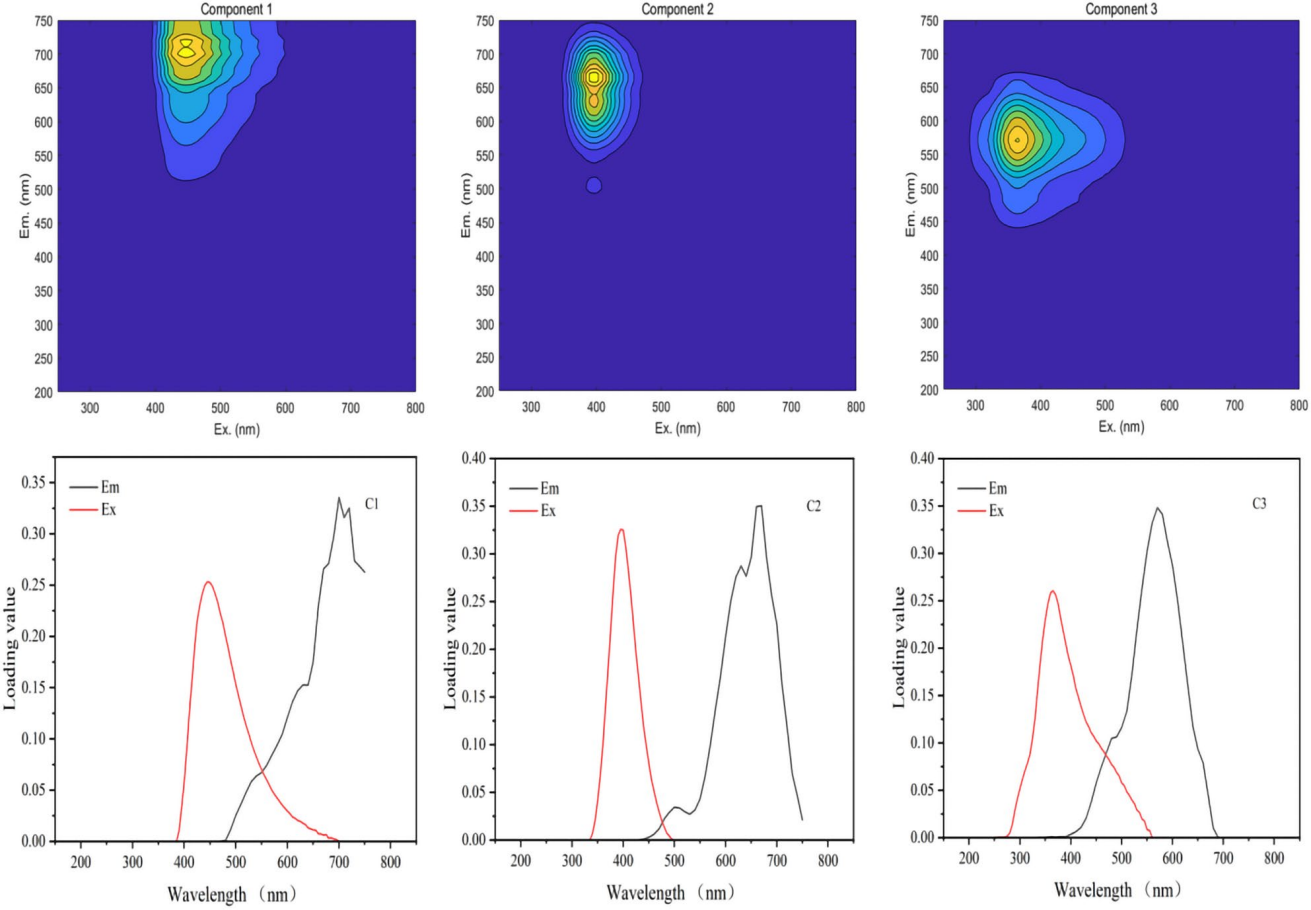
Fluorescence components and loading diagrams of DOM resolved by the PARAFAC model.

Subsequently, the PARAFAC model was used to analyze the spectral information. An outlier test was employed to identify and remove abnormal samples, followed by a residual analysis and outlier correction to determine the number of factors. Model analysis determined the optimal number of factors to be three. Three distinct fluorescence components in the samples were identified using the split-half test and random consistency analysis. Comparison with available fluorescent fractions in the literature using the OpenFlour online database revealed that Component 2 (C2, Ex/Em = 400/670 nm) and Component 3 (C3, Ex/Em = 365/570 nm) were consistent with lipid-like and humic-like compounds, respectively (with a similarity > 90%). Component 1 (C1, Ex/Em = 450/700 nm), which has not been previously reported, was presumed to represent an unknown humic substance (Fig. 16).

**Figure 17:**
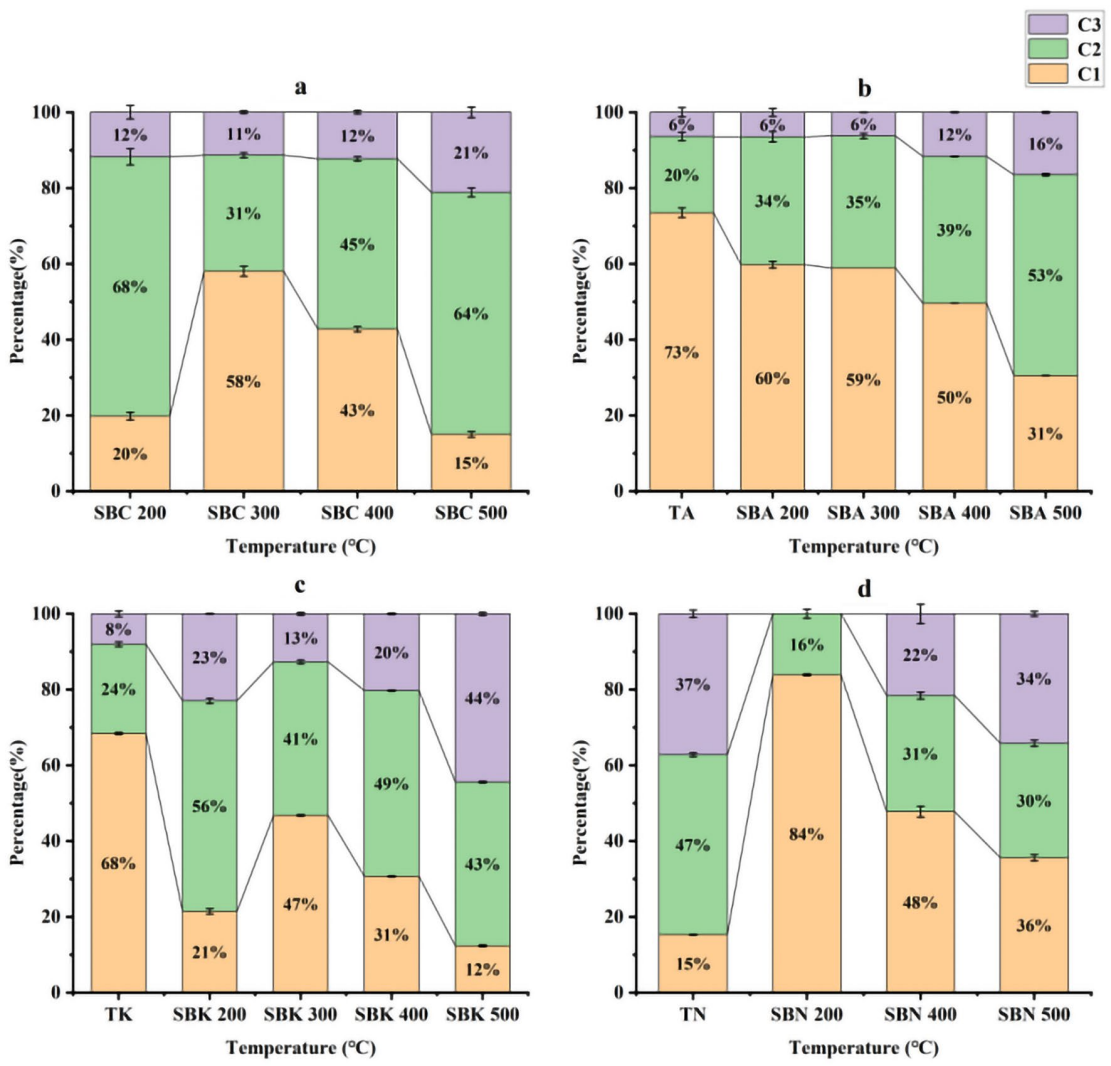
Percentage distribution of three fluorescent components in samples DOM.

The influence of different modifiers and preparation temperatures on the proportion of the three fluorescent components in DOM is depicted in Fig. 17. Regarding the effect of modifiers, it is evident that Na_2_CO_3_ and K_2_CO_3_ primarily released fluorescent substances resembling C1, whereas NaOH had an impact on the proportion of C2 and C3 components (Fig. 17, TA, TK, TN). This can be attributed to the corrosive nature of strong bases^32–34^.

In the context of the effect of preparation temperature, it becomes apparent that when the biochar preparation temperature was below 200 °C, the DOM in the SBA and SBK groups was dominated by the release of C1 substances. Conversely, the SBC and SBN groups were characterized by the prevalence of lipid-like compounds and humic substances. However, when the preparation temperature exceeded 300 °C, the proportion of C1 components in the DOM of each group began to decrease, and the predominant fluorescent substances shifted towards lipids and humic substances. This shift also indicates an increase in humic substances with increasing temperature (Fig. 17^35^).

The results of UV spectral values and fluorescence analysis revealed that the source of DOM of Snow Lotus medicinal residues biochar is mainly the external environment. It has been shown that DOM from this source is mainly composed of humus, carbohydrates and proteins^16,36^, and it was further found in this study that high temperature and modifiers increase the degree of DOM decay. In this study, it was further found that high temperature and modifiers increase the degree of DOM decay and decrease the molecular weight, which indirectly unclogs the micropores of the biochar. It could catalyze the adsorption process of the biochar^37^.

## Conclusion

The modifier can enhance the yield of biochar derived from medicinal residues. Spectral characterization using NIR and FT-IR revealed differences in the effects of various modifications on the medicinal residues and biochar. Our study found that both NaOH and Na_2_CO_3_ positively influenced the alteration and stability of aromatic groups in biochar. Opposite effect on the aromatization of biochar DOM, This led to a greater stability in the molecular weight of DOM Increased decay of DOM prompts the release of large amounts of humic substances and lipid-like fluorescent substances. The release of exogenous biochar biochar DOM indirectly unclogs the pores of biochar, which can expanding the contact area of biochar with pollutants. These are pivotal factors contributing to the enhanced adsorption capacity of modified charcoal.

In assessing the overall impact of each modifier on the structural changes in both biochar and DOM, it is evident that Na_2_CO_3_ is more conducive to stabilizing the biochar structure. Moreover, it offers economic and environmental benefits compared with NaOH, as it reduces the risk of secondary pollution resulting from DOM release from biochar. Therefore, it is more appropriate to choose Na_2_CO_3_ as the modifier of snow lotus residues biochar to further study its adsorption performance.

## Data Availability

The datasets generated during and/or analyzed during the current study aavailable from the corresponding author on reasonable request.
